# Sedation practices in South African ICUs: results of a national survey

**DOI:** 10.1186/cc14577

**Published:** 2015-03-16

**Authors:** F Paruk, S Chetty

**Affiliations:** 1 University of Witwatersrand, Johannesburg, South Africa

## Introduction

There has been a paradigm shift in the approach to sedation of critically ill patients. The purpose of this study was to evaluate current sedation-related practices in South African ICUs.

## Methods

A validated questionnaire was distributed electronically to physician members of various medical databases in South Africa as South Africa does not have a formal registry of critical care practitioners.

## Results

One hundred and twenty-six of 174 respondents indicated that they practice in the ICU setting. Sixty-six per cent were specialists and mainly anaesthesiologists (42%), whilst 32% were critical care subspecialists. The public and private-sector representation was 64% and 46% respectively. A written sedation guideline is implemented by 42%. Forty-three per cent utilise a sedation scale, with the Ramsey Sedation Scale being the commonest in use. However, 38% of sedation scale users do so infrequently. Daily interruption of sedation is practiced by 75%. Light sedation is targeted by 42% and 14% do not follow any sedation targets. Upon admission and on subsequent days, sedation targets are achieved most of the time by 48% and 69% of the respondents respectively. Whilst a wide variety of sedatives are prescribed, midazolam constitutes the most commonly prescribed agent. Dexmedetomidine is the agent of choice for postcardiac surgery patients with cardiovascular comorbidities, delirious patients, during weaning and for non-invasive ventilation. Propofol is the agent of choice amongst neurological patients. The respondents indicated that there is a need for local sedation guidelines.

## Conclusion

The findings are comparable with reports of sedation surveys conducted in other countries. There is an evidence-practice gap that needs to be addressed.

